# Differential effects of BCG vaccine on immune responses induced by vi polysaccharide typhoid fever vaccination: an explorative randomized trial

**DOI:** 10.1007/s10096-020-03813-y

**Published:** 2020-02-17

**Authors:** Bastiaan A. Blok, Rob J.W. Arts, Reinout van Crevel, Peter Aaby, Leo A.B. Joosten, Christine S. Benn, Mihai G. Netea

**Affiliations:** 1 Department of Internal Medicine and Radboud Center for Infectious Diseases (RCI), Radboud University Medical Center, 6526 GA Nijmegen, The Netherlands; 2 Research Center for Vitamins and Vaccines, Bandim Health Project, Statens Serum Institut, DK-2300 Copenhagen, Denmark; 3 Odense Patient Data Explorative Network, University of Southern Denmark/Odense University Hospital, DK-5000 Odense, Denmark

**Keywords:** BCG, Typhoid fever vaccine, Heterologous effects

## Abstract

The Vi polysaccharide typhoid fever vaccine (TFV) provides incomplete protection against typhoid fever. BCG, the vaccine against tuberculosis, can potentiate immune responses to other vaccines through induction of trained innate immunity and heterologous adaptive immunity. We performed an explorative, randomized, noncontrolled open trial to investigate whether BCG vaccination increases humoral and cellular response to TFV and whether BCG and TFV modulate nonspecific immune responses. Thirty volunteers were randomized to receive either TFV alone or BCG followed by TFV after 2 weeks. Ex vivo leukocyte responses and anti-Vi IgG antibody titers were measured 2 weeks and 3 months after TFV. BCG administration prior to TFV vaccination did not increase specific humoral or cellular immune responses to *Salmonella typhi*. TFV vaccination decreased pro-inflammatory responses to non-related stimuli. This effect was counteracted by prior BCG administration, which also led to decreased IL-10 and increased IL-22 responses to non-related stimuli. In an in vitro model of trained immunity TFV led to immunotolerance, which was partially reversed by BCG-induced trained immunity. BCG does not modulate adaptive immune responses to TFV but partially prevents inhibition of innate immune responses induced by TFV. Nonspecific effects of vaccines to unrelated microbial stimuli must be considered in the evaluation of their biological effects (ClinicalTrials.gov NCT02175420).

**Electronic supplementary material:**

The online version of this article (10.1007/s10096-020-03813-y) contains supplementary material, which is available to authorized users.

## Introduction

Typhoid fever is a significant health problem in low-income countries and in returning travelers in high-income countries [1–3]. There are two available vaccines for protection against infection with *Salmonella typhi (S. typhi)*: the live attenuated oral Ty21a vaccine, containing the attenuated Ty21a strain of *S. typhi,* and the non-live parenteral Vi capsular polysaccharide vaccine, a subunit vaccine made from purified Vi capsular polysaccharide (typhoid fever vaccine; TFV). Several trials have shown suboptimal efficacy of these vaccines, with the Ty21a and Vi polysaccharide vaccines conferring 3-year cumulative protective efficacies of 48% and 55%, respectively [4]. Whereas the oral Ty21a vaccine confers protection by inducing both antibody formation and cell mediated immune responses, protection against infection with *S. typhi* after Vi polysaccharide vaccination is thought to be generated mainly by formation of anti-Vi specific IgG antibodies [5, 6].

*Bacille Calmette-Guérin* (BCG) vaccine is used for the protection against tuberculosis. Apart from its protective effect against tuberculosis, BCG has also been shown to confer protection against mortality and morbidity due to all-cause mortality in low-birth weight children [7]. The mechanism behind this nonspecific effect of BCG vaccination is hypothesized to involve the induction of memory properties of the innate immune system, also known as trained immunity and induction of heterologous lymphocyte responses [8, 9]. BCG vaccination of adults as well as children leads to increased ex vivo cytokine responses to non-mycobacterial antigens several weeks after vaccination [10, 11]. BCG has also been shown to increase adaptive antibody response to concurrent or subsequent vaccinations, such as hepatitis B vaccine, pneumococcal vaccine, and influenza vaccine [12–14].

Considering these beneficial nonspecific effects of BCG vaccination, we hypothesized that BCG may potentiate the induction of innate and/or adaptive immune responses induced by the Vi capsular polysaccharide *Salmonella* vaccine. The present study has three aims: first, to investigate whether BCG vaccination increases the adaptive immune response to Vi polysaccharide vaccine; second, to investigate whether TFV modulates the innate immune response to heterologous (i.e., non-*Salmonella*) microbial ligands (innate immune memory); and third, to assess whether BCG can modulate these potential nonspecific effects induced by TFV vaccine.

## Methods

### Study design

**Fig. 1:**
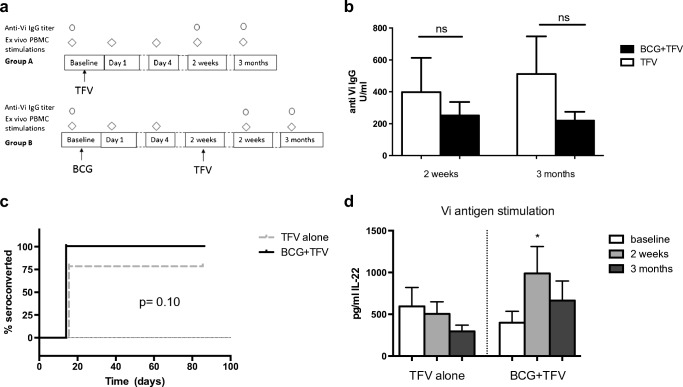
**a** Overview of study procedures. **b** Absolute anti-Vi IgG antibody titer at 2 weeks and 3 months after TFV in subjects vaccinated with either TFV alone or BCG prior to TFV. Mann-Whitney U-test of antibody levels at the same time point between groups. **c** Rate of seroconversion in subjects vaccinated with either TFV alone or BCG prior to TFV. Fisher’s exact test. *N* = 29; * *p* < 0.05. **d** Ex vivo IL-22 production of PBMCs stimulated with Vi antigen. Wilcoxon signed-rank test

This study was an explorative, single-center, randomized, noncontrolled open trial performed from September 2013 to July 2015. Healthy adult volunteers with no history of prior BCG or TFV vaccination, and no known pathology at the time of inclusion, were eligible to participate. Subjects using medication and those who lived in *S. typhi* endemic areas were not eligible for participation. Informed consent was signed, and subjects were randomized in order of enrollment by alternating assignment to receive TFV alone (group A), or to receive BCG followed 2 weeks later by TFV (group B). Blood was drawn before, 1 day (24 h ± 5 h) and 4 days after the first vaccination (BCG for group A; TFV for group B) and subsequently 2 weeks and 3 months after TFV (Fig. 1a). The protocol was approved by the Arnhem-Nijmegen Ethical Committee, and the study was conducted in accordance of the declaration of Helsinki. Subjects who were eligible to receive BCG and TFV due to work or travel in endemic countries as well as normal healthy volunteers were included.

At first, 20 subjects were included between February 2014 and May 2014 and followed for the study period after which data was analyzed. Since we set up this trial as an explorative study, a borderline significant result on seroconversion prompted us to include ten additional subjects from February 2015 to April 2015, of whom one was excluded after day 1. The exact same experiments were performed in the additional volunteers. Data for 29 available subjects are presented. The trial was registered at clinicaltrials.gov (NCT02175420).

Participants were vaccinated by experienced nurses at the Radboud Travel Clinic (Radboudumc, Nijmegen, The Netherlands). All participants were vaccinated with Typhim Vi (Sanofi Pasteur, SA) using a standard dose of 0.5 ml intramuscularly containing 0.05 mg/ml purified Vi capsular polysaccharide of *S. typhi* Ty2 strain. Participants randomized to receive BCG vaccine were vaccinated with BCG Danish 1331 (Staten Serum Institut, Denmark) using a standard dose of 0.1 ml intradermally containing between 2 million and 8 million viable CFU per ml.

### PBMC isolation and stimulation

PBMCs were isolated using density-gradient separation over Ficoll-Paque (GE Healthcare, UK**).** Briefly, whole blood was diluted 1:1 with PBS, and PBMCs were separated using Ficoll. Cells were washed three times with cold PBS and resuspended in RPMI-1640 (Invitrogen) supplemented with gentamycin (50 μg/ml), glutamax (2 mM), and pyruvate (1 mM). Cells were counted using a Coulter counter and adjusted to 5 × 10^6^/ml. A total number of 5 × 10^5^ PBMCs in an end volume of 200 μl were added to 96-well round-bottom plates (Corning). Cells were incubated at 37 °C and 5% CO2 for 24 h (TNF-α, IL-1β, IL-6), 48 h (IFN-γ, IL-10), or 7 days (IL-17, IL-22) after which supernatants were collected and stored at −20 °C until analysis. Ten percent human pool serum was added for the 7-day stimulation assays. PBMCs were cultured with RPMI (negative control), LPS (10 ng/ml, Sigma-Aldrich), heat-killed *Staphylococcus aureus* (1 × 10^6^/ml; *S. aureus*), heat-killed *Candida albicans* conidia strain UC 820 (1 × 10^6^/ml; *C. albicans*), sonicated *Mycobacterium tuberculosis* H37Rv (10 μg/ml; *M. tuberculosis*), heat-killed *Escherichia coli* (1 × 10^6^/ml; *E. coli*), or Typhim Vi antigen (0,005 mg/ml, Sanofi Pasteur). Human pool serum was obtained from the hematology department of Radboudumc, Nijmegen.

### Assessment of serological response to typhoid fever vaccination

Before, 2 weeks and 3 months after typhoid fever vaccination, blood was collected for serology. Serum was stored at −20 °C until analysis. Anti-Vi IgG antibody levels were measured using ELISA (VaccZyme Salmonella Typhi Vi IgG kit, The Binding Site, Birmingham, UK) according to instructions of the manufacturer. Antibody titers are expressed as U/ml.

### ELISA measurements of cytokines

Cytokines were measured in supernatants of stimulated PBMCs using commercially available ELISA kits from R&D Systems, USA (IL-1β, TNF-α, IL-22, IL-17), or Sanquin, The Netherlands (IL-6, IL-10, IFN-γ) according to the manufacturers’ instructions. All samples from one subject were measured on the same ELISA plate to avoid artifacts from batch variation between ELISA plates.

### In vitro trained immunity model

To investigate the effects of BCG and TFV in vitro, a trained immunity model was used as previously described [10, 15]. Buffy coats from healthy donors were obtained after written informed consent (Sanquin blood bank, Nijmegen, The Netherlands). PBMCs were isolated using density-gradient separation over Ficoll-Paque as described above. Monocytes were obtained by density-gradient separation over Percoll, followed by 1 hour of adherence to 96-well flat bottom culture plates (Corning), after which cells were washed once and cultured for 24 h with either BCG 10 μg/ml (SSI, Denmark), Typhim Vi (0,5 μg/ml; Sanofi Pasteur), a combination of both or culture medium (RPMI 1640 supplemented with gentamycin, GlutaMAX and pyruvate) in an end volume of 200 μl. After 24 h of stimulation at 37 °C and 5% CO2, cells were washed once with warm PBS, and RPMI supplemented with 10% human pool serum was added to an end volume of 200 μl. At day 6, cells were cultured with culture medium, LPS (10 ng/ml; Sigma-Aldrich, St. Louis, MO, USA) or Pam3Cys (10 μg/ml EMC Microcollections, Tuebingen, Germany) for 24 h at 37 °C and 5% CO2 after which plates were centrifuged and supernatants were stored at −20 °C until cytokines were measured. LPS and Pam3Cys were used since they have been tested in previous studies using this model.

### Statistical analyses

Differences in cytokine production and antibody titers between study groups were compared with a T-test or Mann-Whitney U-test depending on the distribution of the data. Differences in the proportion of subjects that reached seroconversion in the different study groups were compared using Fisher’s exact test. To assess the effect of BCG and TFV on ex vivo cytokine production, data before and after vaccination were compared using a paired T-test or Wilcoxon signed-rank test depending on distribution of the data. Results are described as cytokine production to a certain stimulus before vaccination compared to cytokine production to the same stimulus at different time points after vaccination. Data was analyzed using GraphPad Prism, version 6.0 (GraphPad Software, San Diego, California). A *p* value of <0.05 was considered statistically significant. Since this was an explorative study, no correction for multiple testing was performed. All data are expressed as mean ± SEM unless stated otherwise.

## Results

### Characteristics of study population and side effects

A total of 30 volunteers were randomized for vaccination, 15 to TFV (group A) and 15 to BCG followed by TFV (group B). One subject from study group A was excluded after day 1 because of a vaccination history that included previous TFV. The majority of subjects were female (group A: 12/14; group B: 13/15); median age was similar in both groups (21 years, range 20–27). All participants in the BCG group reported erythema, minor swelling, and tenderness at injection site, which resolved within 1 month. After TFV vaccination, only transient pain at the injection site was reported.

### Prior BCG vaccination does not increase adaptive response to TFV

#### Humoral antibody response

To investigate the potentiating effect of BCG vaccination on the antibody response to TFV, we measured absolute IgG antibody titers to *Salmonella* Vi antigen at baseline, 2 weeks and 3 months after TFV, as well as the proportion of subjects with seroconversion (defined as a fourfold increase in antibody titer compared to baseline) at 2 weeks and 3 months after TFV in both groups. Baseline levels of anti-Vi IgG were either very low or undetectable in both groups, with no difference between the groups. The absolute anti-Vi IgG titer 2 weeks and 3 months after TFV was higher in subjects who did not receive prior BCG, although this effect was not statistically significant and mainly caused by one very high responder in the TFV alone group (Fig. 1b).

There was no significant difference in seroconversion at 2 weeks in subjects that received BCG prior to TFV compared to those who only received TFV; this was seen in 15/15 in the BCG + TFV groups versus 11/14 in the TFV alone group (Fig. 1c). This effect was sustained at the 3-month follow-up time point (Fisher’s exact test, *p* = 0.10).

#### Typhoid fever specific cytokine responses

We assessed whether TFV or BCG induced short-term T cell responses and trained immunity by assessing cytokine production capacity to either TFV or unrelated stimuli. Cytokine responses after PBMC in vitro stimulation with TFV were poor, with no induction of innate cytokines (TNF-α, IL-6, IL-1β, IL-10) or adaptive cytokines IFN-γ or IL-17 (data not shown). BCG prior to TFV led to a significant increase in IL-22 production 2 weeks after TFV, but this effect was not sustained at 3 months (Fig. 1d).

### Short-term modulation of adaptive and innate immune responses by TFV and BCG vaccination

**Fig. 2:**
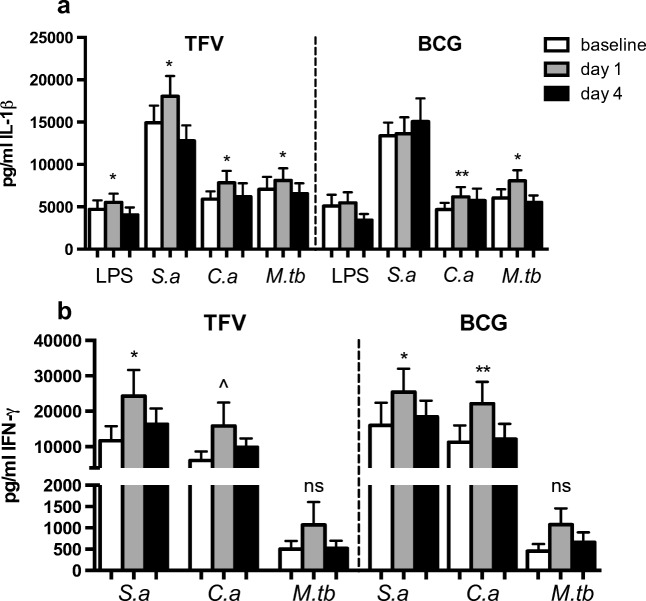
Ex vivo production of IL-1β (**a**) and IFN-γ (**b**) by PBMCs stimulated with LPS, heat-killed *S. aureus* (*S.a*), heat-killed *C. albicans* (*C.a*), and sonicated *M. tuberculosis* (*M.tb*) at baseline, and at 1 and 4 days after vaccination with TFV or BCG vaccine. Wilcoxon signed-rank test comparing values before and after vaccination with the same stimulus; N = 29, ^ *p* = 0.06, * *p* < 0.05, ** *p* < 0.01

TFV vaccination increased pro-inflammatory cytokine production (IL-1β, IFN-γ, and TNF-α, IL-6) upon stimulation with LPS, *S. aureus*, *C. albicans*, and *M. tuberculosis* 1 day after vaccination (Fig. 2a, b and supplementary Fig. 1A, B) compared to before vaccination. This increase in cytokine production returned to baseline level 4 days after vaccination. In contrast, no effects were observed on IL-10, IL-17, or IL-22 production (supplementary Fig. 1C–E). After BCG vaccination production of IFN-γ, IL-1β, and IL-6 to heterologous stimuli at day 1 was increased compared to before vaccination, which returned to baseline at day 4 (Fig. 2a, b and supplementary Fig. 1A, B), although the effects did not reach statistical significance in about half of the cytokine-antigen combinations. No effects were observed on TNF-α, IL-10, IL-17, or IL-22 production (supplementary Fig. 1C–E).

### TFV induces long-term innate immune tolerance, which is partially reversed by BCG

**Fig. 3:**
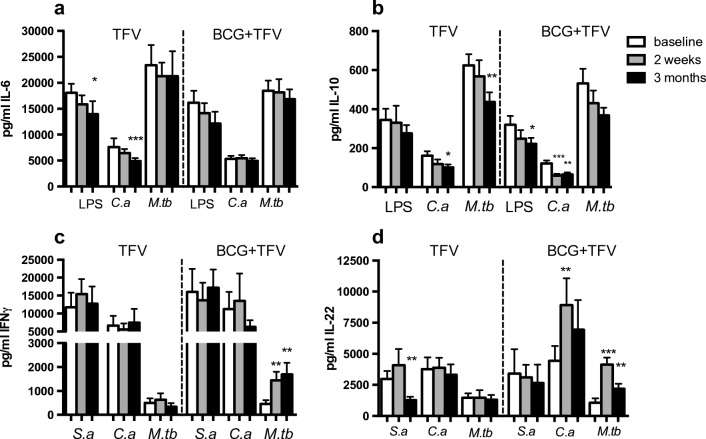
Ex vivo production of IL-6 (**a**), IL-10 (**b**), IFN-γ (**c**), and IL-22 (**d**) by PBMCs stimulated with LPS, heat-killed *S. aureus* (*S.a*), heat-killed *C. albicans* (*C.a*), and sonicated *M. tuberculosis* (*M.tb*), before TFV or BCG vaccination (baseline) and at 2 weeks and 3 months after TFV vaccination. Wilcoxon signed-rank test comparing values before and after vaccination with the same stimulus; N = 29, * *p* < 0.05, ** *p* < 0.01, *** *p* < 0.001

We assessed long-term induction of trained immunity by comparing cytokine responses to unrelated microbial ligands at baseline to responses 2 weeks and 3 months after TFV vaccination. TFV vaccination was associated with significantly decreased TNF-α, IL-1β, and IL-6 responses upon stimulation with LPS, as well as significantly decreased IL-6 production to stimulation with *C. albicans*: a de facto induction of immune tolerance (Fig. 3a and supplementary Fig. 2A, B)*,* although it was striking that TNF-α production to *C. albicans* was upregulated 2 weeks after vaccination. TNF-α and IL-1β production upon stimulation with *S. aureus* and *M. tuberculosis* were also decreased 3 months after vaccination, but this effect was not statistically significant (supplementary Fig. 2a, b). IL-6 production to *S. aureus* was not affected (data not shown). The production of the anti-inflammatory cytokine IL-10 was decreased at 3 months after TFV, and for stimulation with *C. albicans* and *M. tuberculosis*, this reached statistical significance (Fig. 3b). No effects were observed on IFN-γ and IL-17 responses, but IL-22 production upon stimulation with *S. aureus* was significantly decreased 3 months after TFV vaccination (Fig. 3c, d, supplementary Fig. 2C).

Individuals who received BCG prior to TFV vaccination showed partial abrogation of reduction in production of TNF-α, IL-1β, and IL-6 following TFV vaccination (Fig. 3a, supplementary Fig. 2A, B). BCG also counteracted inhibition of IL-6 production to *C. albicans* stimulation following TFV vaccination. Interestingly, BCG administered prior to TFV led to significant decreases in IL-10 production to LPS, *S. aureus*, and *C. albicans* at 2 weeks and 3 months after TFV vaccination (Fig. 3b). As expected, subjects who received BCG prior to TFV showed significantly increased IFN-γ and IL-22 production to *M. tuberculosis* 2 weeks and 3 months after TFV (Fig. 3c, d). Strikingly, BCG prior to TFV led to significantly increased IL-22 responses to stimulation with *C. albicans* and *E. coli* and abrogated the decrease in IL-22 production to *S. aureus* observed in the TFV alone group (Fig. 3d and supplementary Fig. 2D).

### TFV induces immune tolerance in an in vitro model

**Fig. 4:**
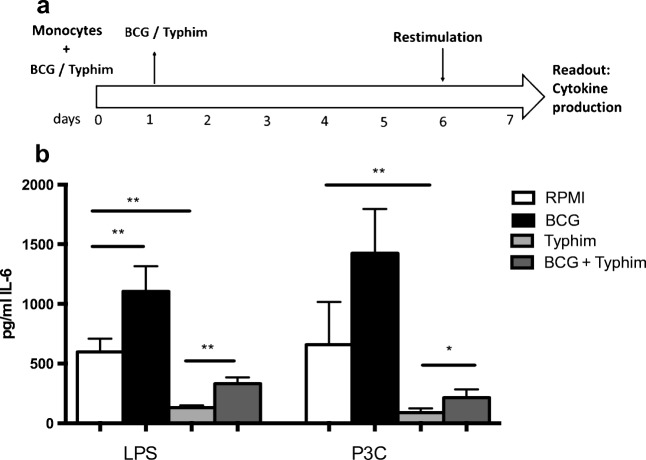
**a** In vitro model of trained immunity. **b** Monocytes were primed with RPMI, BCG, Typhim Vi, or BCG + Typhim Vi for 24 h. Stimuli were washed off, and cells were kept in culture for 6 days, after which they were restimulated with LPS or Pam3Cys for 24 H*. IL*-6 was measured in supernatants. Wilcoxon signed-rank test, *N* = 8, * *p* < 0.05, ** *p* < 0.01

To further validate the immunomodulatory effects of TFV, we used a well-defined in vitro model of trained immunity (Fig. 4a). When monocytes were primed for 24 h with TFV and rechallenged with TLR4 ligand LPS or TLR2 ligand Pam3Cys 1 week afterwards, IL-6 production was significantly decreased. Interestingly, when BCG was added to TFV during the first 24 h of priming, this effect was partially reversed (Fig. 4b), in line with the effects observed in the clinical trial.

## Discussion

We investigated whether BCG vaccination increases the humoral and cellular response to TFV and whether BCG and TFV modulate the immune response to non-related antigens.

BCG vaccination prior to TFV did not affect absolute *S. typhi* antibody titers, and although seroconversion was more common in the BCG group, this failed to reach statistical significance. We did observe a transiently increased cellular recall IL-22 response to TFV, although it should be noted that due to the 2-week interval between the two vaccines and the known kinetics of immune responses elicited by BCG, this might have been an effect purely elicited by BCG without it affecting the response to TFV. A similar trend to improved seroconversion to influenza vaccine after previous BCG vaccination was observed in an earlier study from our group [14]. BCG has been shown to affect innate and T cell-mediated immune responses to protein, peptide, or conjugated polysaccharide vaccines, which behave as T-dependent antigens [12–14]. In contrast, BCG might not affect the response to TFV, a capsular polysaccharide vaccine, which acts as a T cell-independent antigen [16]. A second possibility is that the batch of BCG used in this study was less immunogenic compared to batches used in previous studies, as batch-to-batch variation in immunogenicity and capacity for inducing trained immunity has been observed previously [17].

In addition to specific induction of lymphocyte responses, vaccines also modulate nonspecific innate immune responses [10, 11]. In this study BCG and TFV induced transient, short-term increased pro-inflammatory cytokine production to unrelated stimuli. This effect probably reflects an acute-phase reaction, since trained immunity needs days to weeks to be established [18].

On a longer term, 2 weeks and 3 months after TFV vaccination, innate pro-inflammatory cytokine production to LPS was decreased: a de facto innate immune tolerance effect. This effect was abrogated when BCG was given prior to TFV. Furthermore, in these subjects, we observed a decrease of the anti-inflammatory cytokine IL-10 to stimulation with LPS, suggesting that BCG skews the balance towards a more pro-inflammatory response to LPS, although TFV may also have contributed to this effect. It is likely that this opposing effect of BCG is due to its capacity to induce trained immunity and thus counteract immune tolerance [19]. Another possibility is that viable BCG was present at the time of TFV vaccination and that the ongoing immune response modulated the immune-tolerizing effect of TFV. Since inflammatory monocytes are important in the initial immune response to *S. typhi* [20], it could be speculated that BCG vaccination would lead to a more efficient immune response to typhoid fever. Future studies are warranted to investigate the effects of BCG vaccination on the incidence or outcome of typhoid fever.

This may be the first study describing heterologous effects of the Vi polysaccharide typhoid vaccine in a human in vivo setting, but there are earlier data to support two possible explanations for this immunosuppressive effect of TFV. First, earlier studies found that Vi polysaccharide inhibits monocyte and T cell activation, leading to reduced heterologous cytokine responses [18, 21]. Since we observed immunosuppressive effects of TFV in an in vitro model of trained immunity, it most likely has a direct effect on monocyte function. Second, the immunomodulatory effect of TFV may be mediated by sialylated IgG antibodies, which are induced upon immunization with T cell-independent antigens and have been shown to have immunosuppressive actions [22].

An immunosuppressive effect of the non-live TFV is in line with previous immunological studies that have shown immunosuppressive effects of the inactive influenza vaccine [14], MVA [23], and diphtheria-tetanus-pertussis vaccine [24]. These effects are in contrast to the immune-stimulatory effects observed after the live BCG [10] and smallpox vaccines [23]. In parallel, an increasing number of epidemiological studies from low-income settings show that non-live vaccines, while protective against the target disease, may increase mortality from non-related causes [25, 26], while live vaccines reduce overall mortality more than can be explained by target disease prevention [7, 27]. While simplistic, it is tempting to speculate that the immunosuppressive and immune-stimulatory effects observed after vaccination with non-live and live vaccines, respectively, explain why the vaccines seem to have so different effects on overall mortality in real life.

We did not find increased pro-inflammatory cytokine responses to unrelated antigens, also known as trained immunity, in subjects that received BCG prior to TFV, in contrast to previous studies [10, 11, 28]. This might be explained by batch variation in the BCG vaccine [21], or due the fact that the immune-tolerizing effect of TFV opposed the innate training effects of BCG. Although we did not find increased pro-inflammatory cytokine responses, we observed reduced production of IL-10, suggesting that the overall balance of the immune response is more pro-inflammatory. This is in line with a previous study which found decreased IL-10 responses after BCG vaccination in low-birth weight infants [11]. Furthermore, we found heterologous effects of BCG on T cell immunity in the form of increased IL-22 responses to *E. coli* and *C. albicans*, in line with previous studies of BCG showing strong effects on heterologous IL-22 responses [28, 29].

Several limitations to our study should be noted. With initially ten subjects per study group of TFV alone or BCG + TFV, the sample size is small. Ten additional volunteers (five individuals/groups) were recruited after observing a potential effect on seroconversion. However, the final conclusion after analyzing the data in the entire group of vaccinated individuals was that BCG did not impact the serological effects of typhoid fever vaccine. Another limitation of the study is that the reported *p* values were not corrected for multiple testing, due to the explorative nature of this study.

In conclusion, BCG vaccination does not enhance immunogenicity of TFV vaccination, but larger studies are needed to examine a possible moderate increase in antibody responses. The Vi polysaccharide typhoid fever vaccine has nonspecific immuno-tolerizing effects in vitro and in vivo, which are partially abrogated by prior BCG vaccination. This finding supports the concept that both live and non-live vaccines have important nonspecific immunomodulatory effects, which influence the immune response to other microorganisms and may influence overall morbidity and mortality.

## Electronic supplementary material

ESM 1 (PDF 507 kb)

## Abbreviations

BCG: Bacillus Calmette-Guérin
LPS: Lipopolysaccharide
MVA: Modified Vaccinia Ankara
Pam3Cys: Pam3Cys-Ser-Lys(4)
PBMC: Peripheral blood mononuclear cell
PBS: Phosphate-buffered saline
TFV: Typhoid fever vaccine
