# Supplementary material for: Differential effects of BCG vaccine on immune responses induced by vi polysaccharide typhoid fever vaccination: an explorative randomized trial
Source: Eur J Clin Microbiol Infect Dis. 2020 Feb 17;39(6):1177–84. doi: 10.1007/s10096-020-03813-y (PMC7225183; doi:10.1007/s10096-020-03813-y)
Supplement: Supplementary file 1 — (PDF 507 kb) [file 10096_2020_3813_MOESM1_ESM.pdf]

SUPPLEMENTARY FIGURE 1

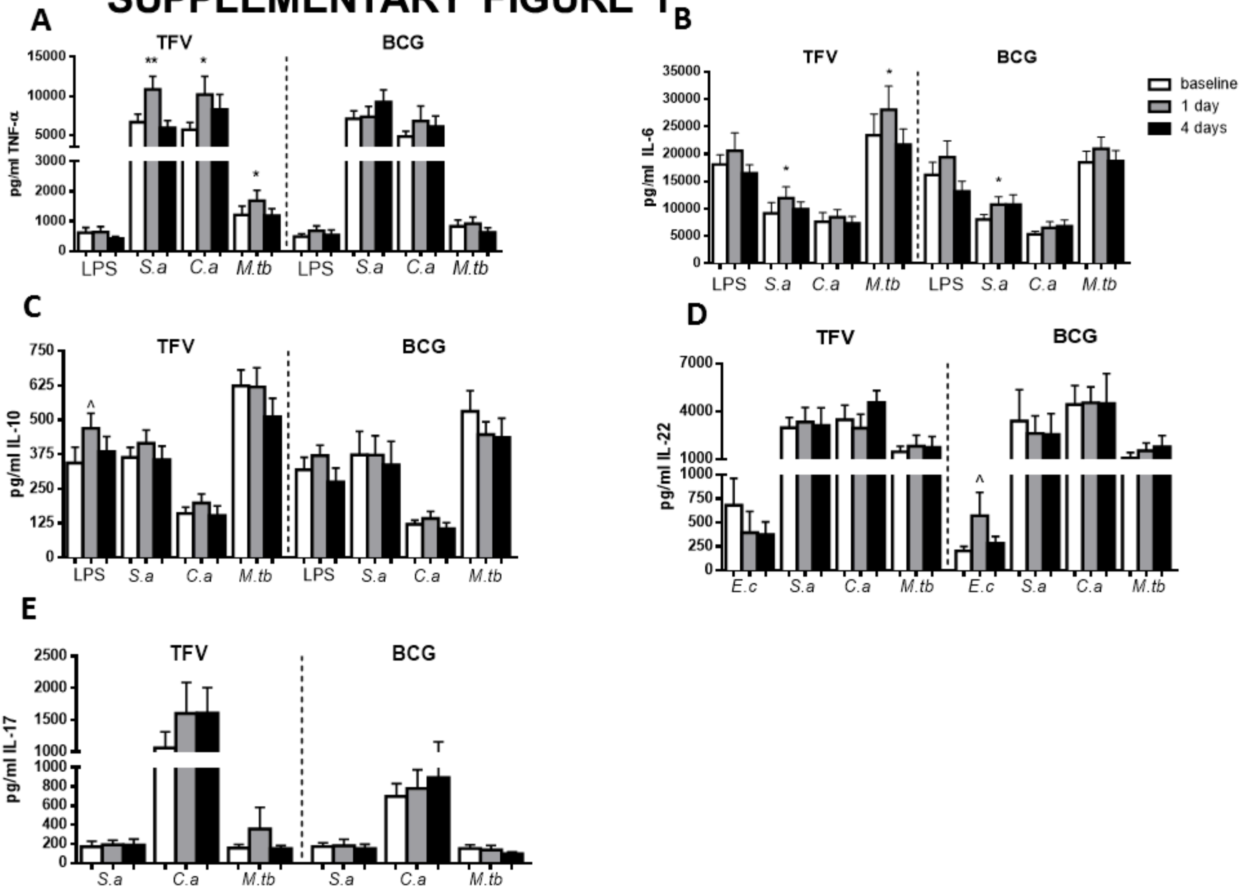

Supplementary figure 1

Ex vivo production of TNF-α (A), IL-6 (B), IL-10 (C), IL-22 (D) and IL-17 (E) by PBMCs stimulated with LPS, heat killed *S.aureus (S.a)*, heat-killed *C.albicans (C.a)*, sonicated *M. tuberculosis (M.tb)* and heat-killed *E. coli (E.c)* at baseline, and at 1 and 4 days after vaccination with TFV or BCG vaccine. Wilcoxon signed-rank test. N=29, ^ p=0.06, * p<0.05, ** p<0.01.

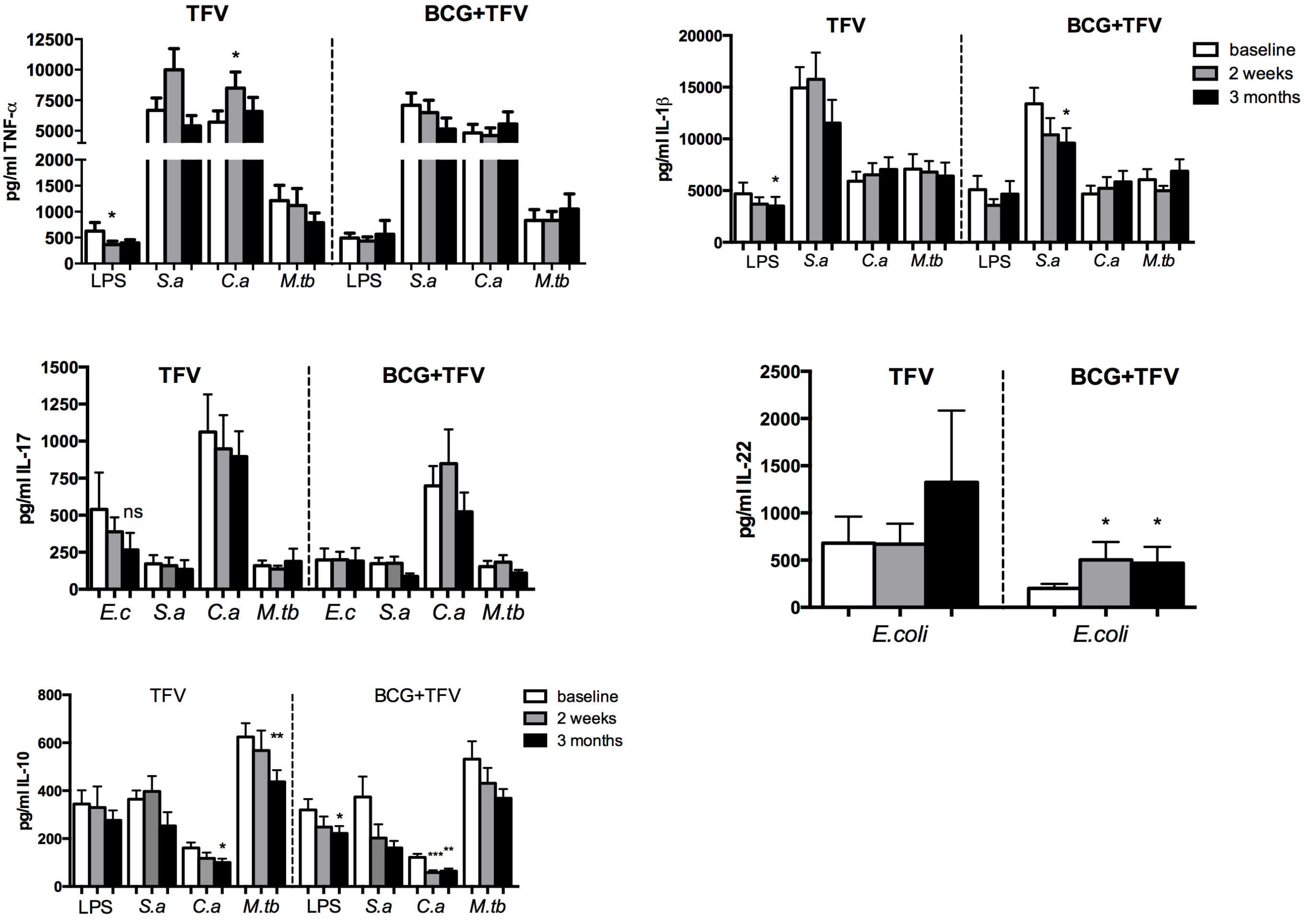

Supplementary figure 2

Ex vivo production of TNF-α (A), IL-1β (B), IL-17 (C) and IL-22 (D) by PBMCs stimulated with LPS, heat-killed *S. aureus (S.a)*, sonicated *M. tuberculosis (M.tb)* or *E. coli (E.c)*, before BCG or TFV vaccination (baseline) and at 2 weeks and 3 months after TFV vaccination. Wilcoxon signed-rank test. N=29, * p<0.05.

CONSORT

TRANSPARENT REPORTING of TRIALS

CONSORT 2010 Flow Diagram

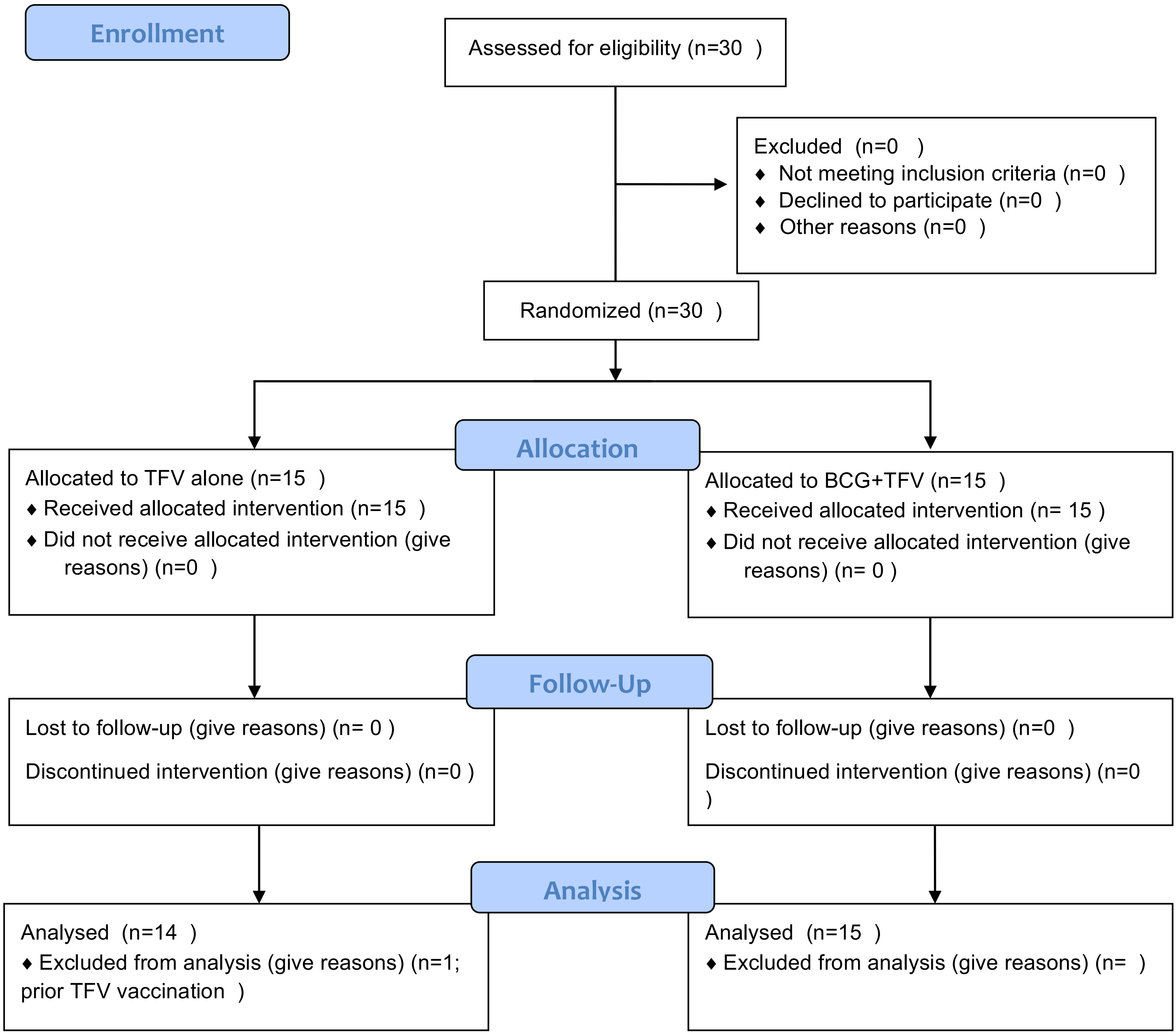

Supplementary figure 3.

CONSORT diagram.
